# Nonmedical Prescription Opioid Use among a Sample of College Students: Prevalence and Predictors

**DOI:** 10.3390/pharmacy9020106

**Published:** 2021-05-28

**Authors:** Lisa L. Weyandt, Bergljót Gyda Gudmundsdottir, Emily Shepard, Leslie Brick, Ashley Buchanan, Christine Clarkin, Alyssa Francis, Marisa Marraccini

**Affiliations:** 1 University of Rhode Island, Kingston, RI 02881, USA; emilys2618@uri.edu (E.S.); buchanan@uri.edu (A.B.); chrisclarkin@uri.edu (C.C.); alyssa_f@uri.edu (A.F.); 2 University of Iceland, 107 Reykjavík, Iceland; bgg@hi.is; 3 Brown University, Providence, RI 02912, USA; leslie_brick@brown.edu; 4 University of North Carolina at Chapel Hill, Chapel Hill, NC 27599, USA; mmarracc@unc.edu

**Keywords:** prescription opioid, college students, substance misuse

## Abstract

Nonmedical use of prescription opioid medication (NMPO) in the United States is a public health crisis, resulting in high rates of emergency room visits, morbidity, and mortality. The purpose of this study was to explore prevalence estimates and correlates of NMPO among a convenience sample of college students in the northeast and southeast regions of the US to help generate directions for future research. Motivations for misuse, age of onset, access, concomitant substance use, and individual factors were investigated among a sample of undergraduate students from two universities. Participants (*N* = 847) completed a battery of various self-report measures. Findings revealed that 7.7% (Southeastern University) and 12.8% of students (Northeastern University) reported lifetime NMPO, whereas past-month NMPO was reported by 0.8% and 0.9% of participants, respectively. Lifetime history of regularly using alcohol, nonmedical use of benzodiazepine medication, nonmedical use of prescription stimulants, symptoms of depression and anxiety, and executive functioning (i.e., metacognition and behavioral regulation) were significantly related to lifetime history of NMPO in this college sample. These findings offer several potential subsequent lines of investigation regarding the associations between various demographic and psychological factors and NMPO. Future research is needed to help identify college students who are at risk of NMPO.

## 1. Introduction

Misuse of prescription drugs is defined as the use of prescription medications without a valid prescription or in a manner other than prescribed. Misuse can include nonmedical use, medical misuse, the use of another person’s prescription medication, or the misuse of one’s own prescription [1,2]. Nonmedical use of prescription opioids (NMPO) is of significant concern because of the possibly dangerous health outcomes associated with misuse, as well as the tendency for NMPO to transition to use of illicit opioids, including heroin [3]. Currently, all classes of opioids represent a particularly problematic prescription drug given their high rates of use, addiction, and overdose [4,5]. In particular, trends demonstrate that, in the last decade, NMPO and related negative outcomes have risen. For example, Gladden and colleagues reported a 90% increase in opioid-related overdoses from 2013–2017 [6], while the CDC reported a 10.6% increase in deaths specifically involving prescription opioid overdose between 2015 and 2016 [7]. More recently, the CDC reported 70,980 overdose deaths in the United States in 2019, a 4.6% increase from the previous year, including 50,042 deaths involving opioids [8]. Regarding misuse, in 2017, the National Survey on Drug Use and Health reported that 11.4 million people had misused opioids in the past year, with 1.7 million people misusing prescription opioids [9,10]. While the surge of NMPO that occurred in the early 2010s has since flattened, prescription and synthetic opioid misuse remain a significant concern among college students because of the increased risk of developing opioid use disorder and overdose associated with these drugs [5,11].

College students between the ages of 18–25, known to engage in high rates of substance use, including alcohol and cannabis, have also been found to engage in misuse of prescription medications, including NMPO [12,13,14]. Specific prevalence estimates of NMPO within college student samples are highly variable, ranging from between 7.5% to 32% across samples [15,16,17,18,19,20]. Although results from a national sample of full-time university students revealed that NMPO peaked between 2003 and 2006, these rates have been maintained since this time, with 6.6% of the college-age population reporting misuse [21]. The college population is of particular concern because it has been demonstrated that college students are at greater risk for sharing and improperly storing medications [22], indicating that prescription medication misuse is probable, and studies are needed to explore NMPO in college students.

### 1.1. Motivations for Prescription Opioid Misuse

Factors associated with an increased likelihood of NMPO, in general, include physical pain, anxiety, depression, executive functioning deficits, and other comorbid substance misuse [23,24,25,26,27]. A primary motivation for use of prescription opioids among the general adult population is both physical and emotional pain relief [24]. Individuals with chronic pain appear to be at greater risk for NMPO, because of greater access to opioids for medical use and due to illegal means that may be taken when medical treatment is perceived as inadequate for pain relief [26]. Research supports a high rate of prescriptions in medical contexts for pain management [28,29] and, concomitantly, a strong relationship between prescribing patterns for prescription opioids and overdose mortality rates [30,31,32]. Martin and colleagues reported that after 12 weeks of prescription opioid use, 50% of adults continued to use opioids for over a year, even when the initial 12 weeks of prescription opioid use followed a physician’s instructions [33].

### 1.2. Psychological and Neuropsychological Factors

Other psychological factors related to pain have been identified as predictors of NMPO. Specifically, among patients with chronic occupational musculoskeletal disorders, anxiety and depression predict a higher risk for NMPO [23]. Similarly, among adults with moderate or severe pain, pain-related anxiety was significantly related to several indicators of NMPO, including self-reported opioid addiction, family concerns due to opioid use, history of opioid detoxification, and the number of opioid-related problems [34]. The intensity of pain has not, however, been found to be predictive of increased NMPO, but negative affect and catastrophizing along with the presence of pain are predictive of misuse [25]. Thus, pain and corresponding emotional states have a powerful association with NMPO and necessitate further study.

In addition to anxiety, depression is related to prescription opioid use in the presence of pain and independent of pain in the general population [23,25,27]. In the presence of chronic pain, depression may contribute to further risk of NMPO, with a particular risk of intentional overdose (i.e., suicide) [4].Independent of pain, suicide-related thoughts and behaviors are also related to NMPO, with individuals, particularly college students, citing management of suicidal and depressive feelings as a primary motive for misuse [35]. More specifically, in a large sample of college students reporting recreational NMPO in the past six months, 56% met the criteria for Major Depressive Disorder [36]. These studies support a relationship between depression and NMPO and warrant further investigation. The present study seeks to expand on such findings by exploring whether subthreshold symptoms of anxiety and depression are also related to NMPO among college students.

Although compromised emotional well-being appears to increase the likelihood of NMPO, cognitive factors may also play a role. In particular, executive functioning (EF), i.e., a set of consciously controlled cognitive processes that include planning and goal-oriented behavior [37], has been studied extensively concerning substance use. Studies have consistently demonstrated that impairments in EF are associated with increased substance use, including alcohol, nicotine, prescription stimulants, cocaine, and other illicit drugs [38,39,40,41,42,43]. Among college students, EF deficits are strong predictors of engagement in risky behavior and substance use [27]; however, little is known about EF as a predictor of NMPO among this population. Specific aspects of EF, namely, emotion dysregulation [44] and poorer metacognition [45], have been demonstrated to be related to NMPO, with reduced metacognition appearing among individuals with long-term patterns of opioid misuse [45]. The results of the present study may shed light on whether deficits in metacognition and emotion regulation are associated with NMPO.

### 1.3. Comorbid Substance Use

A particularly dangerous comorbidity to NMPO may include the use of other substances, as the harmful effects of opioid misuse are exacerbated by comorbid use of other drugs and alcohol [46,47]. Concerning college students, several studies have found that this population reports combining prescription opioids with alcohol, marijuana, benzodiazepines, prescription stimulants, and other types of substances [13,48]. Comorbid use of benzodiazepines and opioid medication is of particular concern, as benzodiazepines are associated with a high risk of dependence, misuse, and overdose [49]. This behavior poses a significant public health concern on multiple levels: increased health risks of combining prescription opioids with other substances, illegal activities to procure opioids, physiological withdrawal symptoms, social problems, poorer academic performance, and higher rates of college dropout [50,51]. Investigation of comorbid substance use with prescription opioids is warranted given the risks of such behavior and the commonality of comorbid misuse.

### 1.4. Individual Factors

Although psychological variables are critical in helping to identify specific risk factors regarding NMPO, variability in risk according to individual factors is also important to consider. Despite being limited in scope and number, several studies have suggested that specific individual factors appear to be related to NMPO among college students, including GPA, impulsivity, levels of stress, Greek membership, and perceived harm of these medications [16,52,53]. Gender differences have also been explored in NMPO. Although findings from most studies suggest that males are more likely to misuse prescription opioids, in light of some studies finding no gender differences [52,54], additional research is merited [55]. Concerning differences according to race and ethnicity, several studies have reported that the prevalence of NMPO is higher among white college students than students of other racial and ethnic backgrounds; however, questions remain regarding NMPO among college students from minoritized backgrounds [56,57,58]. Finally, age of onset is another covariate that must be examined to appropriately target intervention programs, as preliminary research supports that earlier onset of substance use is related to the development of opioid use disorder later in life [59].

The present study sought to expand existing knowledge in the literature concerning NMPO by exploring the prevalence and psychological correlates of this behavior in a sample of college students with three main aims. First, the study assessed the previous (i.e., lifetime) and past-month prevalence of NMPO among college students located in two regions of the USA (northeast and southeast regions of the USA). Lifetime and past 30-day misuse were selected as these intervals are commonly used in the research of opioid misuse, and it has been demonstrated that past misuse is predictive of subsequent misuse [58,59,60,61]. Accordingly, the study explored the prevalence of students reporting lifetime and past 30-day NMPO.

Second, the study explored the associations between a variety of health risk behaviors, including physical pain, concomitant substance use, sexual risk behavior [62], depression and anxiety symptoms, executive functioning, demographic factors (e.g., race, ethnicity, and gender), and NMPO. Specifically, it was hypothesized that (a) students reporting higher levels of anxiety, depression, and executive dysfunction would be more likely to report NMPO; (b) students endorsing greater pain behaviors in their activities would be more likely to endorse NMPO; (c) male students would be more likely to disclose NMPO than female students; and (d) students who reported NMPO would be more likely to report concomitant drug use, specifically alcohol, benzodiazepines, marijuana, and prescription stimulant medication. The third aim of the study was to examine NMPO motives, analyze the main sources for attaining prescription opioids for misuse, and identify specific prescription opioids commonly misused. Findings from this study will help to shed light on characteristics of college students who may have increased likelihood of NMPO and possibly comorbid substance use. The findings will also help to generate designs and hypotheses for future research.

## 2. Materials and Methods

### 2.1. Participants

**Figure 1:**
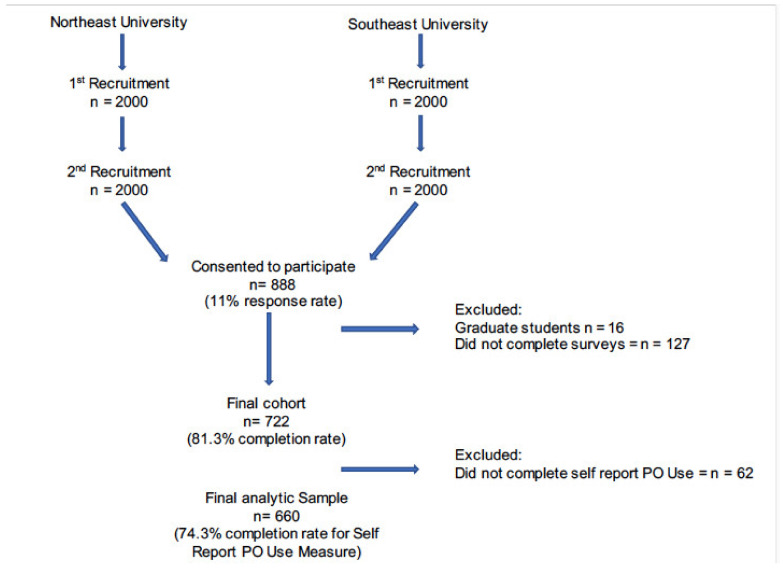
Recruitment procedures.

Undergraduate students were recruited from two universities located in the United States. Recruitment of participants occurred during the spring 2018 semester. The study was reviewed and approved by the two universities’ Institutional Review Boards. The registrar’s office generated a random sample of students (*n* = 2000) at each university, with a target enrollment of 500 students per school (for a total *n* = 1000). Students were contacted via email and invited to participate in the study. No directly identifiable information was gathered from participants; however, given the nature of the study, a certificate of confidentiality was attained to protect participant data. Enrollment was ongoing throughout the semester, and a second wave of recruitment occurred during the semester, including an additional randomly selected sample (*n* = 2000) from each university (see Figure 1).

A total of 888 participants (89% of targeted enrollment) consented to participate in the study, resulting in an overall response rate of 22%. Because this study focused on undergraduate students and NMPO, students who did not report year in school (*n* = 25) and students who reported attending graduate school (*n* = 16) were excluded. The final sample included *N* = 847 undergraduate students.

### 2.2. Procedures

All participants completed informed consent before responding to measures made available through REDCap, an electronic data collection system [63]. Participants completed a battery of self-report measures designed to assess medical and nonmedical prescription opioid use, engagement in risky health behaviors (e.g., sexual activity and other drug use), psychological functioning (e.g., depressive symptoms and anxiety symptoms), physiological functioning (i.e., pain behaviors), and neuropsychological functioning (i.e., executive function). Only those measures relevant to this study are reported here. Upon completion of the study, participants were provided an opportunity to enter a drawing for one of 10 $50 gift cards.

### 2.3. Measures

#### 2.3.1. Demographic Variables

Participants were instructed to answer questions related to demographic characteristics, including sex (defined as the sex participants were assigned on their original birth certificate), gender identity (which included choices for male, female, or transgender and not identifying as female, male, or transgender), race (American Indian or Native Alaskan, Asian, black or African American, Native Hawaiian or Other Pacific Islander, white, or other), ethnicity (Hispanic or Latino, not Hispanic, unknown, or choose not to answer), age, and year in college. Questions also related to the individual factors of grade point average (GPA) and association with Greek life (yes/no). Possession of a current or previous lifetime prescription for opioid medication was measured with two dichotomous (yes/no) questions asking participants “Do you have a prescription for opioids?” and “Have you ever been prescribed opioids?”. To capture lifetime history of prescription opioid misuse, participants were asked, “Have you ever used your prescription for reasons other than prescribed (e.g., a prescription for hip pain used for back pain, for psychological stress, to get high) or in higher amounts than prescribed?”

#### 2.3.2. Prescription Opioid Use

The principal variable of interest for this study was nonmedical use of prescription opioids [1]. To capture NMPO, participants were asked “have you ever used prescription opioids non-medically in your lifetime?”, with the following definition of non-medical use included: “non-medical use is defined as use without a valid prescription or use with a prescription in a manner other than prescribed, i.e., in higher dosage, for different reasons, or during a different time period than prescribed.”

#### 2.3.3. Motivations for Use

Motives for opioid use were measured with the Opioid Prescription Medication Motives Questionnaire (OPMMQ) [64]. The OPMMQ is a five-point Likert scale designed to measure how often individuals use pain medication based on 28 different motivational reasons such as “because it is fun” and “to decrease anxiety”, with answers ranging from “never” to “very often” [65]. The motivational reasons fit within a 4-factor model including pain, social, enhancement, and coping, with each factor supporting differential patterns of association between prescription opioid-related variables. Although exploratory and confirmatory factor analyses (CFA) support a 4-factor model [66], the current study examined individual items due to the small number of students endorsing NMPO (*n* = 68), precluding a CFA in the current sample.

#### 2.3.4. Frequency of Use

Frequency of NMPO was measured using the Nonmedical Use of Prescription Opioids Questionnaire (NUPOQ). The NUPOQ is a self-report survey designed to measure prevalence and frequency of current and lifetime drug use. The survey includes nonmedical use of prescription opioids and stimulants, heroin, and other drugs such as cocaine, marijuana, hallucinogens, and alcohol [65]. Response choices include “no”, “yes”, “not applicable”, “don’t know” and, when directed, an open-ended response column to indicate the number of months or days drug use occurred. This measure has been used in research related to health risks and NMPO among young adults [65].

Participants were led through a series of questions dependent upon endorsed drug use. Respondents were initially asked about NMPO and the use of heroin in separate questions phrased, “Have you ever used [drug] in your lifetime?”. NMPO was defined to participants as “use without a valid prescription or use with a prescription in a manner other than prescribed, i.e., in a higher dosage, for different reasons, or during a different time period than prescribed.” Participants were also provided a list of examples including, immediate-release oxycodone (e.g., Roxicet, Roxicodone, and Percocet), extended-release oxycodone (e.g., Oxycontin), hydrocodone (e.g., Vicodin and Lorcet), oxymorphone (e.g., Opana and Numorphan), morphine (e.g., MS Contin, Oramorph), hydromorphone (e.g., Dilaudid), fentanyl (e.g., Duragesic and Actiq), buprenorphine (e.g., Suboxone and Subutex), methadone codeine (e.g., Tylenol 3 and 4), and tramadol (e.g., Ultram, Tramal). Participants were then asked, “What other drugs have you used in your lifetime?” and instructed to select all that applied, including those of interest in this study (e.g., marijuana, benzodiazepines, and prescription stimulants). Examples of benzodiazepines (i.e., Xanax, Klonopin, Librium, and Valium) and prescription stimulants (i.e., Adderall, Ritalin, and Concerta) were provided to participants for reference.

Lifetime drug and alcohol use variables were measured as a binary response of yes or no, with “regular alcohol use,” defined as 3 or more times a week. When answering “yes”, past-month opioid use was measured by the number of days in the past 30 days the opioids were used, including the average number of pills and the milligrams of each pill.

Finally, one item from the NOPUQ was used to determine the most common prescription opioids misused among participants. The item specifically asked, “Which prescription opioids did you take nonmedically?”. Participants were instructed to choose “all that apply” from an extensive list of prescription opioids, with the option to select “other” and specify.

#### 2.3.5. Prescription Source/Age of Misuse

Participants were asked several follow-up questions regarding prescription source and age of onset of use. Specifically, participants were asked “Where did you obtain the prescription opioids you used? (check all that apply).” Options included: “Primary care MD”, “Urgent care”, “Emergency room”, “Orthopedic MD”, “Pain Management Specialist”, “Dentist”, “Specialist Other”, “Friend(s)”, “Parent(s)”, “Bought Illegally”, “Stole”, or “Other”. Age of misuse was determined from a final set of questions that first prompted participants to report if misuse began “before starting college” or “after starting college” to measure age of onset. Participants were next asked, “How old were you when you misused opioids?” Response options included, “younger than 15 years old”, “Between the ages of 15 and 18”, and “Older than 18 years old”.

#### 2.3.6. Behavior Rating Inventory of Executive Function—Adult

The BRIEF-A is a 75-item questionnaire composed of two indices, metacognition and behavior regulation, in which participants are asked about their executive functioning in daily activities, with “never”, “sometimes”, or “often” as response options. The Metacognition Index (MCI) comprises five subscales: initiating, working memory, plan/organize, task monitoring, and organization of materials. The Behavior Regulation Index (BRI) constitutes four subscales: inhibition, shifting, emotional control, and self-monitoring [66]. Together, the MCI and BRI form a Global Executive Composite (GEC) score, representing an overall appraisal of executive functioning, with higher scores indicating greater executive dysfunction. Studies suggest adequate to excellent internal consistency for BRIEF-A subscales and BRI, MCI, and GEC indices [66,67]. We included MCI (30 items) and BRI in the current study, with adequate internal reliability for each subscale, respectively (α = 0.930; 0.956).

#### 2.3.7. Sexual Risk Survey

The SRS is a 23-item questionnaire where respondents are asked to report the frequency with which they have participated in each of a range of sexual risk behaviors during the preceding 6 months, using open-ended responses. Turchik and Garske previously reported adequate internal consistency (0.90) for the full scale, and the Cronbach’s alphas for four of the five subscales, (1) sexual risk taking with uncommitted partners, (2) risky sex acts (3) impulsive sexual behaviors, and (4) intent to engage in risky sexual behaviors, are adequate as well (0.90, 0.82, 0.79, and 0.81, respectively). The internal consistency of the fifth subscale, risky anal sex acts, however, was poorer at 0.63 [68].

To calculate frequency distributions as described by Turchik and Garske [69], responses were coded into ordinal categories, ranging from 0 to 100%. Responses of zero were retained, and additional responses were recoded according to frequency: 1 = 40%; 2 = 41–70%; 3 = 71–90%; and 4 = 91–100%. Results of the confirmatory factor analyses and internal reliability estimates reported previously for this sample demonstrated adequate fit for a five-factor model and adequate estimates for four of the five factors (α = 0.778–0.930) [68]. Because reliability estimates for anal sex (α = 0.655) remained low, this subscale was excluded from the analyses.

#### 2.3.8. DSM-5 Self-Rated Level 1 Cross-Cutting Symptom Measure—Adult

The DSM-5 Level 1 Cross-Cutting Symptom Measure is a self-rated measure that assesses mental health domains that are important across 13 psychiatric domains, including depression, anger, mania, anxiety, somatic symptoms, suicidal ideation, psychosis, sleep problems, memory, repetitive thoughts and behaviors, dissociation, personality functioning, and substance use. Respondents were asked to rate how much (or how often) the individual has been bothered by the specific symptom during the past 2 weeks. The measure has been found to be clinically useful and to have good test-retest reliability in the DSM-5 field trials that were conducted in adult clinical samples across the United States and in Canada [68,69]. For the current study, a mean score for depressive symptoms that included two items (e.g., “feeling down, depressed, or hopeless”) and anxiety symptoms that included three items (e.g., “avoiding situations that make you anxious”) were calculated. Both subscales demonstrated adequate internal reliability (α = 0.817 and 0.843, respectively).

#### 2.3.9. PROMIS^®®^ (Patient-Reported Outcomes Measurement Information System)

The PROMIS measures encompass various mental and physical health outcomes among children, youth, and adults, created and developed by the National Institutes of Health (NIH), with studies supporting their reliability and validity [70,71,72]. PROMIS Items are presented as statements (e.g., “When I was in pain I moved extremely slowly”), or as questions (e.g., “How intense was your average pain?”), to which responses are provided on a Likert scale (e.g., “had no pain”/“never” to “always”, or “had no pain” to “very severe”). In the present study, the PROMIS^®®^ v1.1 Item Bank–Pain Behavior-Short Form 7a was administered to participants. Item scores were summed for an overall pain behavior score converted to a *T*-score, with a mean of 50 and a standard deviation of 10, per scoring guidelines.

### 2.4. Statistical Analyses

Descriptive statistics were calculated using SPSS version 26 (IBM, 2018) and logistic regression analyses were conducted using MPlus version 8 to account for the clustering of students across schools using sandwich estimators to compute standard errors (i.e., TYPE = COMPLEX with CLUSTER = school) [73,74]. All continuous variables were assessed for normality before analysis, finding that all variables were normally distributed, and a decision to remove lifetime history of heroin use was made due to its low prevalence (only two students reported a lifetime history of heroin). Demographic (e.g., sex, race, and ethnicity) comparisons were examined across universities to better understand the study sample regarding key demographic characteristics and key variables used in subsequent analyses, including prevalence estimates of NMPO and predictor variables (i.e., drug use and alcohol use).

To address the first aim of the study, identifying the number of students reporting past month and lifetime NMPO, prevalence of NMPO was calculated. Prevalence of sources and type of drug were also calculated.

To address the second aim, a model was used to identify the salient factors related to NMPO. Following bivariate correlational analyses of all variables of interest with lifetime history of NMPO, multiple logistic regression analyses were conducted to examine whether the following self-reported variables were associated with lifetime history of NMPO: Greek status (yes/no), lifetime use of alcohol and drugs commonly used by college students (including marijuana and nonmedical use of benzodiazepine or stimulant medication), metacognition, behavioral regulation, depression, anxiety, and pain behavior. The model also controlled for age, year in college, GPA, sex, race (minority/non-minority ethnic-backgrounds and White students), and ethnicity, but no other variables. To account for the clustering of students within universities (i.e., non-independence of outcomes among students), sandwich estimators were used to compute standard errors [74].

Finally, given the limited information available concerning types of prescription opioids obtained, motivations for misuse, and sources of prescription opioids, we aimed to better characterize the subset of individuals who reported having ever engaged in NMPO. To address this aim, descriptive statistics about sources for obtaining drugs, commonly used drugs, and motives for NMPO are presented.

Missing data in the analytic sample (*n* = 845 undergraduate students) pertaining to the measures included in the study addressing primary aims were minimal for most measures, except for anxiety and depression, ranging from 0% (age and sex) to 43.4% (depressive and anxiety symptoms). Missing data were handled during model estimation with Full Information Maximum Likelihood (FIML), a robust approach for handling data that are missing at random (MAR) or missing completely at random (MCAR).

## 3. Results

Across both universities, participants were, on average, 20 years of age (*SD* = 1.28), predominantly white (83.2%), and female (71.2%) (see Table 1). Note that one student identified as transgender, and three students endorsed the item “I do not identify as female, male, or transgender.” At the southeast university, the percentage of participants reporting their sex assigned at birth as female was significantly higher compared to that of the northeast university. Compared to the northeast university, a higher percentage of participants from the southeast university reported their race as Asian or “other”, whereas the reverse was true for participants reporting their race as White. Additionally, a significantly higher percentage of participants from the southeast university reported being of sophomore status compared to the northeast university.

### 3.1. NMPO Characteristics

Prevalence estimates of key study variables are presented separately for each university in Table 1 and Table 2. Nearly 11% of students reported NMPO at least once during their lifetime across both universities, with significantly higher estimates reported at the northeast college (12.8%) compared to the southeast college (7.7%). Estimates of past-month prevalence of NMPO were much lower, with less than 1.0% of students at each university (*n* = 3, southeast; *n* = 4, northeast) endorsing NMPO. Note that a total of 20 participants selected an answer other than “Yes” or “No” to the question “Have you ever used prescription opioids non-medically in your life?” (“Not Applicable”, *n* = 5; “Don’t Know”, *n* = 14; and “Refuse to Answer”, *n* = 1). Bivariate analyses of percentages revealed a statistically significant group difference of lifetime NMPO across male (15%) and female (10%) participants (*z* = 2.07, *p* = 0.04). Approximately one-third of the participants at each university reported a lifetime history of having a prescription for opioids; note, however, that very few students reported having a current prescription for opioid medication (1.8% of the entire sample). Lifetime history of regularly drinking alcohol, using marijuana, benzodiazepine medication, and prescription stimulant medication differed according to university, with estimates being significantly higher at the northeastern compared to the southeastern university for all alcohol and drug use histories.

### 3.2. Correlates of Nonmedical Prescription Opioid Use

Bivariate correlational analyses (Table 3) revealed several significant associations between the independent variables of interest (e.g., Greek affiliation, lifetime history of drug and alcohol use, symptoms of depression, executive functioning, and pain behavior), covariates (e.g., age, sex, race, ethnicity, year in college, and GPA), and lifetime history of NMPO (see Table 3). Although measures of sexual risk behavior (i.e., SRS subscales) were intercorrelated, they were not significantly correlated with lifetime history of NMPO. In contrast, Greek status (yes/no), lifetime use of alcohol and drugs (i.e., marijuana and nonmedical use of benzodiazepine or stimulant medication), behavior regulation, metacognition, depression, and pain behavior were significantly correlated with lifetime history of NMPO.

Based on those findings, a logistic regression analysis examining Greek status (yes/no), lifetime use of alcohol and marijuana, nonmedical use of benzodiazepine or stimulant medication, metacognition, behavioral regulation, depression, anxiety, and pain behavior as predictors of lifetime NMPO are shown in Supplementary Table S1. After controlling for demographic characteristics (sex, race, ethnicity, age, GPA, and year in college), reporting of lifetime history of regularly using alcohol (adjusted odds ratio (aOR) = 1.78, 95% confidence interval (CI): 1.46–2.17), nonmedical use of benzodiazepine medication (aOR = 6.76, 95% CI; 3.72–12.28), nonmedical use of prescription stimulant medication (aOR = 2.77, 95% CI: 2.20–3.50), metacognition (aOR = 0.89, 95% CI: 0.88–0.90), behavioral regulation (aOR = 1.30, 95% CI: 1.00–1.67), depression (aOR = 1.44, 95% CI: 1.34–1.55), and anxiety (aOR = 0.830, 95% CI: 0.743–0.927) were significantly related to reports of lifetime history of NMPO.

### 3.3. Characteristics of Students Reporting NMPO

#### 3.3.1. Age of Onset

Among students reporting a lifetime history of NMPO and answering the question about age of onset of NMPO use, 47.7% reported first trying prescription opioids nonmedically before entering college, and 52.3% reported using prescription stimulants for the first time after entering college. The majority of students reporting a history of NMPO and answering the questions about age of onset (*n* = 92) generally reported age of onset to be older than 18 years (43.5% total; 44.0% southeast, 43.3% northeast) or between 15 and 18 years (28.3% total; 36.0% southeast, or 25.4% northeast). Four students reported age of onset to be younger than 15 years (4.3% total; 4.0% southeast, 4.5% northeast).

#### 3.3.2. Sources for Obtaining Prescription Opioids

As shown in Table 4, across both universities, friends (40.0% and 26.9% in the southeast and northeast, respectively) emerged as the most frequently reported source for obtaining prescription opioids for nonmedical use. The second most frequent source included dentists (20.9%) for the university in the northeast and parents (20.0%) or purchasing medication illegally (20.0%) for students in the southeast.

#### 3.3.3. Medication Types

The most frequently misused medications are shown in Table 4 and included immediate-release oxycodone, hydrocodone, and codeine. The most commonly misused medication in the southeastern university was hydrocodone (36.0%). In the northeastern university, the most commonly misused medication was codeine (25.4%). The second most common medication misused in the southeastern university was immediate-release oxycodone (32.0%), while in the northeastern university, the second most commonly misused medications were hydrocodone (23.9%) and immediate-release oxycodone (23.9%). Combining data from both universities, the most common medication type was immediate-release oxycodone (26.1%).

#### 3.3.4. Motivations for Use

The frequencies of different motivations for NMPO among lifetime users are shown in Supplementary Table S2. Across universities, the most frequently endorsed motivations for using prescription opioids included “to relieve physical pain” (63.7% of participants across both universities endorsed having used each of these medications at least once by selecting either hardly ever, sometimes, often, or very often), “because it’s fun” (53.2% endorsed having used hardly ever, sometimes, often, or very often), and “because it gives you a pleasant feeling” or “to get high” (48.1% and 47.5%, respectively, endorsed having used hardly ever, sometimes, often, or very often). The least frequently endorsed motivations, as indicated by a rating of never, included “because you are addicted” (87.2% never), “to counter the effects of other drugs” (84.8% never), “because it’s safer than street drugs” (83.5% never), “for back or neck problems” (76.9% never), “because it improves parties and celebrations” (75.9% never)”, “because you feel more self-confident or sure of yourself” (75.9% never), and “to celebrate a special occasion with friends” (75.9% never).

## 4. Discussion

The purpose of the present study was to explore prevalence estimates and correlates of NMPO among college students and to help generate hypotheses for future research. Motivations for misuse, age of onset, access, concomitant substance use, and individual factors were explored among undergraduate students from two universities located in the United States. College students are of particular concern regarding NMPO given previous studies that have demonstrated that these students have an increased likelihood of misusing general prescription medication, including prescription stimulant medication, benzodiazepines, and opioids [12,13,14,22]. Thus, despite results not being appropriate for drawing conclusions concerning population parameter estimates, the present study contributes interesting information regarding possible characteristics and motivations of college students reporting NMPO, and it can assist future research in designing studies and hypotheses to help identify at risk students (and ultimately targeted interventions).

The first aim of the current study was to explore prevalence estimates of students reporting lifetime and past 30-day NMPO in the northeast and southeast regions of the US. Results revealed that a concerning number of undergraduate college students reported lifetime NMPO, with 7.7% and 12.8% of students endorsing such behavior for the southeastern and northeastern universities, respectively. The most commonly misused medications reported by students in the present study included immediate-release oxycodone, hydrocodone, and codeine, and these findings are consistent with previous studies [19]. Estimates of past-month NMPO were much lower, with approximately 1% of participants. These findings are similar to those reported by Kenne and colleagues [18], who described 9.5% of college students in their sample as reporting lifetime NMPO, as well as those found in a large systematic review of prescription opioid use among college students up until 2019, which found prevalence of misuse between approximately 5–20% [14]. Current findings are notably higher, however, than those found by Harries and colleagues, in which 2.2% of college students in the sample reported NMPO in the past 12 months, while 5.3% reported lifetime NMPO [16]. It is important to note that while both Kenne and colleagues’ [18] and Harries and colleagues’ [16] studies were conducted among a sample of college students at a midwestern university, Harries and colleagues’ study [16] was conducted several years later and had a larger, more representative sample. Geographic location differences may possibly explain these disparate findings [14]. Indeed, in the current sample, a higher number of students from the northeastern university reported lifetime NMPO, compared to their southeastern counterparts. Regarding college students in the US overall, nationally representative data reported by Schulenberg and colleagues (2020) [21] revealed that nonmedical use of narcotics other than heroin (e.g., prescription opioids) among this population decreased between 2014–2019, with prevalence estimates of 1.5% (past year) and 5.8% (lifetime). Taken together, prevalence estimates appear to vary across time periods, sampling methods, and geographic location, among other factors. Prevalence estimates reported by Schulenberg et al. (2020) likely offer the most robust data regarding the prevalence of NMPO within the college student population currently; however, the present study adds valuable information concerning the association between various demographic/psychological factors and NMPO. A limitation of the present study is the reliance on a convenience sample; future investigations with larger, more representative samples across different geographical regions are needed. Ideally, such studies would also include measures pertaining to psychological distress (e.g., depression, anxiety, etc.), executive function, pain, and other risk behavior (e.g., other substance use, sexual risk behavior, etc.), similar to the current study. Other limitations include a possible presence of self-reporting bias, as well as under-completion of the survey by individuals with NMPO history and a possible social desirability bias.

Current findings also suggest that self-reported executive dysfunction (i.e., behavioral regulation) and depression are significantly related to higher odds of misusing prescription opioids. In contrast, and contrary to expectations, poorer metacognition and higher levels of anxiety were associated with *lower* odds of NMPO, while controlling for the effects of other correlates. The reverse was true, however, for results of bivariate analyses, in which anxiety and metacognition problems correlated positively with NMPO. Despite these discrepancies, these findings collectively suggest that executive dysfunction, depression, and anxiety may play a role in NMPO; however, additional studies with larger, more representative samples are warranted to further investigate this potential relationship. It is also important to note that previous research supports that use of prescription opioids may increase the risk of depression; hence, those who seek relief via misusing these medications may inadvertently exacerbate their depression symptoms [18,75]. Similarly, Jacobson and colleagues reported that opioids can also impair executive functions, which in turn can deleteriously affect decision-making processes [76]; however, future work is needed to further investigate these relationships.

Contrary to what was hypothesized, when adjusting for the effects of other variables of interest, students endorsing greater pain behavior were *not* more likely to report NMPO. It is important to note, however, that the percentage of students reporting NMPO was relatively small. Previous researchers have found that when anxiety and pain conditions are concurrently reported by patients, some clinicians, despite FDA black box warnings, prescribe both opioids and benzodiazepines, potentially setting the stage for individuals to misuse opioids [49]. Although speculative and needing further study, the unexpected result of individuals endorsing greater pain behavior may also be a result of improved prescribing practices among physicians and a growing awareness of the danger of prescription opioid medication. Specifically, a recent systematic review by Hopkins and colleagues (2019) has demonstrated that interventions aimed to reduce risks among prescribers have generally been effective at reducing the prescription of opioid medication to manage pain [77]. Other studies, however, have demonstrated that patients in the United States are prescribed opioid medication at significantly higher rates than other countries, specifically Canada, Spain, Italy, Taiwan, Korea, the United Kingdom, and New Zealand [78]. Public perception of opioid medication is another key factor to understanding why students endorsing greater pain behavior were not more likely to report NMPO. It has been demonstrated that many individuals who have a valid prescription for opioid medication do not take the medication out of concerns about risk for opioid use disorder [79].

Although bivariate analyses revealed significantly higher lifetime estimates of NMPO for men (15%) relative to women (10%), this group difference was no longer significant once effects of other factors (i.e., use of alcohol, benzodiazepines, prescription stimulants, executive functioning) were adjusted for the analysis. Although findings concerning gender differences have been inconsistent in the literature [14], the present results are similar to those reported by Elliot and Jones, for example, who found similar patterns of NMPO between men and women; however, their sample was based on the 2014 National Survey on Drug Use and Health and did not target college students [80]. Importantly, the current sample comprised of more females (77.1%) than males, and future studies should try to recruit a sample that is more representative in terms of sex, including students who identify as non-binary and students who are transgender. Although the present study inquired about whether students identify as non-binary or transgender, the small sample size of these groups precluded analyses. Research is sorely needed to investigate potential differences among college students concerning gender identification and misuse of prescription opioids, using larger, more representative sample sizes with adequate statistical power.

Consistent with previous findings concerning NMPO, the present results revealed statistically significant bivariate correlations among several health risk factors and behaviors, including lifetime history of alcohol and drug use, symptoms of depression, and executive dysfunction. In contrast, despite evidence suggesting that sexual risk behavior significantly relates to drug use in general [81], including use of prescription opioids, no significant correlations between risky sexual behavior and NMPO were found [81,82,83].

The hypothesis that students who reported NMPO would be more likely to report concomitant drug use (specifically, alcohol, benzodiazepines, marijuana, and prescription stimulant medication) was partially supported. Results revealed that nonmedical use of benzodiazepine medications, nonmedical use of prescription stimulant medications, and a history of regular alcohol use (i.e., three or more times a week), were significantly related to higher odds of NMPO (Table 3). Previous research has also found that nonmedical use of sedatives and tranquilizers was correlated with NMPO [20]. It is not surprising that past prescriptions for opioid medication would relate to future misuse; however, findings regarding benzodiazepine medications, regular alcohol use and prescription stimulants (i.e., polysubstance use) are troubling and warrant further attention. Although previous research supports higher odds of alcohol use among college students who engage in NMPO [13], more research is necessary to solidify this relationship, as well as to determine the relationship between prescription stimulant misuse and NMPO. It is plausible that use of other prescription drugs ‘normalizes’ drug use behavior, increasing the likelihood of NMPO. Comparatively, the relationship between benzodiazepines and opioid misuse has been examined more closely throughout the literature. Specifically, previous research has suggested that concurrent non-medical benzodiazepine and other opioid use are motivated by a desire to get a greater “high” and decrease symptoms of opioid withdrawal [84].

Friends were the most commonly reported source of prescription opioids, consistent with McCabe et al.’s findings from over a decade ago [19]. Relief from physical pain was the most commonly reported motivation among students reporting a lifetime history of NMPO. Additional motives included to relax, relieve pain and anxiety, for enjoyment, and as a sleep aid. These results are similar to those found in a study by McCabe et al. (2007), in which pain relief, to experience a high, to experiment, to sleep, and to relieve anxiety were most commonly reported [19]. These findings illustrate that similar motives for NMPO among young adults have persisted for a considerable period of time. With consistent findings across 10 years, future research should explore possible screening methods and interventions designed to address these commonly endorsed motives to better identify at-risk students and reduce overall misuse.

Given previous research findings concerning prescription opioid misuse among young adults in conjunction with results of the present study concerning co-occurring opioid misuse with other drugs among college students specifically, additional research across college campuses appears warranted. Although outside the scope of the present study, future research should explore whether intervention programs are needed to help educate college students about the deleterious effects of NMPO and combining opioids with other substances.

Importantly, additional studies are needed to explore potential cultural and diversity findings among students from various backgrounds (e.g., sexual orientations and racial and ethnic differences) and whether NMPO prevalence estimates differ within these groups to help inform design of culturally responsive interventions to reduce NMPO. Future research should also examine misuse among college-related groups such as community college students and trade school students, as well as potential regional differences. Because studies have documented higher estimates of NMPO in North America compared to the rest of the world, and opioid misuse is more prevalent in rural areas than in urban areas in the US [32], research focused on community samples in rural areas is needed. Finally, the present study raises questions about whether symptoms of anxiety may be protective of NMPO. This is in opposition to other research that has demonstrated that anxiety is related to increased NMPO; however, it is important to note that previous studies examined anxiety in the context of pain [23,34]. Future studies should attempt to confirm whether anxiety is only positively associated with NMPO in the presence of pain.

There are several limitations of the present study that need to be taken into consideration. First, the study was based on a survey method, and although significant efforts were made to recruit a random, representative sample, the sample was composed of participants who agreed to participate (convenience sample), and they were largely female and White, thereby limiting analyses concerning gender identification beyond male/females as well as race and ethnicity. Additionally, given a relatively low response rate, students who agreed to participate in the study may not be representative of the college student population at the universities studied or of college students and young adults in general (i.e., selection bias). Furthermore, data were missing from the analytic sample from the measures included in the study, with the most data missing from measures of anxiety symptoms (43.4%). During model estimation, missing data were handled with Full Information Maximum Likelihood (FIML), a robust approach for handling missing data. Further limitations include the cross-sectional, self-report study design and small sample sizes with respect to subgroups of interest, thereby compromising statistical power. Accordingly, the present results should *not* be interpreted as indicative of NMPO prevalence among US college students in general. Longitudinal studies are needed, using a stratified sampling technique to explore patterns of NMPO among college students as they progress through their academic programs. Although a strength of the study is that students from two universities were included, generalizability to other institutions, including community colleges and trade schools, is limited, and future research across multiple private and public universities is warranted. Within the survey, limitations included possible lack of exclusivity in answer choices. For example, a student may not have known whether to select “family” or “stole” as their source for prescription opioid medication if they stole the substance from a family member. This finding is potentially significant because of possible differences in the characteristics of individuals who steal medication compared to those who are given the medication. Similarly, it is unclear whether participants who purchased prescription opioids from friends would endorse “friends” or “bought illegally”. Additionally, the present study did not address participant history of tobacco/nicotine use. Use of tobacco/nicotine is strongly associated with other substance use [85,86,87], as well as EF deficits [38,39,40,41,42,43]. Accordingly, future studies should investigate the role of tobacco/nicotine in NMPO. A final limitation is that the survey questioning medical misuse only addressed higher amounts, not more frequent use than prescribed or non-oral administration.

Despite these limitations, this investigation has several strengths and highlights that a significant percentage (10.9%) of college students sampled reported NMPO for a variety of reasons, with the most common being pain relief. In addition, many who endorsed NMPO also reported a history of other substance use, including alcohol and prescription medications. Based on the finding that approximately half of the students (45.1%) surveyed in the current sample reported NMPO *before* college, these findings suggest that future research should explore whether prevention efforts should be aimed at those who have yet to try prescription opioids but may be at risk, perhaps beginning prevention efforts as early as the ninth grade. This type of approach has been successfully implemented to increase knowledge about opioid misuse; however, more research is necessary to assess the long-term effects of such interventions [88]. Despite the limitations of the present study, the findings can help to inform future research with respect to specific variables to investigate with respect to NMPO among college students. Given that friends were identified as the most common source of medication, future studies should explore this area in more detail.

In conclusion, the present study sought to explore predictors and prevalence estimates of NMPO among college students attending colleges in two regions of the United States and to help generate future directions for research. In particular, this study highlighted the potential role of student demographic factors (e.g., sex, age, race/ethnicity, GPA, etc.), psychological distress (e.g., depression, anxiety), executive function, pain behavior, and other substance use in NMPO, with findings offering several possible lines of investigation for future studies. Given the importance of identifying predictors of misuse, the present study also explored motivations and sources of prescription opioids. Results revealed that 7.7% of students attending a college located in the southeast and 12.8% of students attending a college located in the northeast endorsed NMPO.

When examining sex differences in isolation, male participants were more likely to disclose lifetime NMPO than their female counterparts; however, this group difference was no longer evident once other correlates of NMPO were included in the analyses. Specifically, having a past prescription for opioid medication, nonmedical use of benzodiazepine medications, nonmedical use of prescription stimulant medications, a history of regular alcohol use, executive function deficits, and depression symptoms were related to greater odds of NMPO. Physical pain was the most commonly reported motivating factor for NMPO, followed by recreational uses, to decrease anxiety and tension, and to help with sleep. Given the limitations of the study, however, including the reliance on a convenience sample, additional research is needed to further examine psychological factors associated with NMPO, as well as the relationship between NMPO along with other substances, such as benzodiazepines or stimulants. Studies are also needed to explore NMPO among students from diverse backgrounds (SES, gender identification, race, and ethnicity) and different subgroups of college students, such as specific majors, athletes, or members of Greek life. Lastly, future studies are needed to investigate the educational, social, interpersonal, and physical effects of NMPO among the college student population that will ultimately lead to appropriate interventions to decrease NMPO within the college student population.

## Figures and Tables

**Table 1:** Demographic Characteristics of the Sample.

	College in Southeast	College in Northeast	Total
Variable	*n* (Percent)	*n* (Percent)	*n* (Percent)
Sex assigned at birth *			
Female	246 (75.9)	357 (68.3)	603 (71.2)
Male	78 (24.1)	166 (31.7)	244 (28.8)
Race **			
American Indian or Native Alaskan	0 (0.0)	1 (0.2)	1 (0.1)
Asian *	41 (12.7)	19 (3.6)	60 (7.1)
Black or African American	22 (6.8)	23 (4.4)	45 (5.3)
Native Hawaiian or Other Pacific Islander	1 (0.3)	2 (0.4)	3 (0.4)
White *	231 (74.4)	463 (88.5)	704 (83.2)
Other *	18 (5.6)	15 (2.9)	33 (3.9)
Ethnicity			
Hispanic or Latinx	26 (8.0)	33 (6.3)	59 (7.0)
Not Hispanic or Latinx	277 (88.4)	444 (84.9)	730 (86.2)
Year in College *			
Freshman	58 (17.9)	114 (21.8)	172 (20.3)
Sophomore *	91 (28.1)	98 (18.7)	189 (22.3)
Junior	88 (27.2)	143 (27.3)	231 (27.3)
Senior	80 (24.7)	152 (29.1)	232 (27.4)
Greek Status			
Yes	74 (22.8)	153 (29.3)	227 (26.8)
No	249 (76.9)	367 (70.2)	616 (72.7)
Lifetime Prescription for Opioids			
Yes	104 (32.1)	174 (33.3)	278 (32.8)
No	214 (66.0)	334 (63.9)	548 (64.7)
Lifetime Nonmedical Prescription Opioid Use *			
Yes	25 (7.7)	67 (12.8)	92 (10.9)
No	284 (87.74)	420 (80.3)	704 (83.1)
Past Month Nonmedical Prescription Opioid Use			
Yes	3 (0.9)	4 (0.8)	7 (0.8)
No	265 (81.8)	427 (81.6)	692 (81.7)
	**M (SD): range**	**M (SD): range**	**M (SD): range**
Age * (total *n* = 847)	20.09 (1.28): 18–24	20.28 (1.42): 18–24	20.21 (1.37): 18–24
GPA * (total *n* = 799)	3.38 (0.46): 0.92–4.0	3.1 (0.45): 1.43–4.0	3.33 (0.46): 0.92–4.0

Total numbers and percentages may vary due to missingness in data. * Group difference is significant at the 0.05 level; ** group difference is significant at the 0.01 level.

**Table 2:** Prevalence of drug and alcohol use by university.

	College in Southeast		College in Northeast		Total	
	*n*	%	*n*	%	*n*	%
Lifetime Nonmedical Prescription Opioid Use *	25	7.7	67	12.8	92	10.9
Past Month Nonmedical Prescription Opioid Use	3	0.9	4	0.8	7	0.8
Lifetime Prescription for Opioid Medication	104	32.1	174	33.3	278	32.8
Current Prescription for Opioid Medication	3	0.9	12	2.2	15	1.7
Lifetime History of Regularly Drinking Alcohol **	131	43.1	252	52.7	383	49.0
Lifetime Marijuana Use **	142	43.2	321	62.1	463	54.7
Lifetime Nonmedical Use of Benzodiazapine **	18	5.5	61	11.8	79	9.3
Lifetime Nonmedical Use of Prescription Stimulants **	42	12.8	118	22.8	160	18.9

Yes/no prevalence by demographic within each university. * Group difference is significant at the 0.05 level; ** group difference is significant at the 0.01 level.

**Table 3:** Bivariate correlations (Pearson’s *r*) between all independent and dependent variables.

Variable	1	2	3	4	5	6	7	8	9	10	11	12	13	14	15	16	17	18	19	20	21
Age	1																				
2. Sex	0.071 *	1																			
3. Race	0.006	−0.052	1																		
4. Ethnicity	−0.004	−0.045	−0.123 **	1																	
5. Year in college	−0.026	−0.002	0.000	0.027	1																
6. GPA	0.002	−0.019	−0.085 *	0.070	−0.039	1															
7. Lifetime NMPO	0.012	0.041	−0.090 *	0.018	0.106 **	−0.116 **	1														
8. Greek Affiliation	−0.033	0.015	−0.083 *	0.043	0.081 *	−0.056	0.097 **	1													
9. Lifetime History of Regularly Drinking Alcohol	−0.071 *	0.027	−0.104 **	0.045	0.274 **	−0.137 **	0.216 **	0.239 **	1												
10. Lifetime Marijuana Use	−0.031	0.022	−0.144 **	0.023	0.149 **	−0.122 **	0.172 **	0.132 **	0.426 **	1											
11. Lifetime NMPS	−0.041	0.056	−0.104 **	−0.012	0.129 **	−0.183 **	0.354 **	0.129 **	0.300 **	0.379 **	1										
12. Lifetime NMBM	−0.034	0.022	−0.122 **	−0.027	0.095 **	−0.089 *	0.419 **	0.043	0.224 **	0.259 **	0.468 **	1									
13. DSM total depression symptoms	0.014	0.061	−0.053	−0.065	0.089	−0.001	0.167 **	−0.023	0.082	0.105 *	0.145 **	0.126 **	1								
14. DSM total anxiety symptoms	0.006	0.160 **	−0.082	−0.053	0.108 *	−0.009	0.081	−0.015	0.042	0.128 **	0.117 *	0.126 **	0.698 **	1							
15. PROMIS^®®^ Pain Behavior scale	0.027	0.049	−0.094 *	0.097 *	0.074	0.029	0.096 *	0.014	0.088 *	0.121 **	0.122 **	0.139 **	0.220 **	0.232 **	1						
16. BRIEF-A Behavior Regulation Index	−0.010	0.103 **	−0.069	0.003	0.046	−0.098 *	0.122 **	0.024	0.085 *	0.156 **	0.227 **	0.162 **	0.426 **	0.517 **	0.283 **	1					
17. BRIEF-A Metacognition Index	−0.030	0.054	−0.043	0.008	0.018	−0.170 **	0.108 **	−0.011	0.071	0.157 **	0.215 **	0.162 **	0.406 **	0.413 **	0.225 **	0.818 **	1				
18. SRS Sex with Uncommitted Partners subscale	0.138 **	0.048	0.004	−0.042	0.057	0.013	0.071	0.056	0.022	0.034	0.035	0.086	0.037	−0.004	0.006	0.015	0.057	1			
19. SRS Risky Sex Acts subscale	0.136 **	−0.012	0.087	−0.043	−0.035	0.029	−0.013	−0.010	−0.044	−0.007	−0.071	−0.016	0.041	−0.013	−0.050	0.023	0.014	0.570 **	1		
20. SRS Impulsive Sexual Behavior subscale	0.043	0.111 **	−0.005	−0.082	0.046	0.037	0.033	0.019	0.002	0.014	0.005	0.047	0.068	0.034	−0.034	−0.013	−0.006	0.685 **	0.428 **	1	
21. SRS Intent to engage in Risky Sex subscale	0.078	0.150 **	−0.039	−0.061	0.025	0.006	0.080	0.020	0.007	0.031	0.053	0.056	0.007	0.017	−0.024	−0.039	−0.056	0.608 **	0.329 **	0.760 **	1

BRIEF-A: Behavior Rating Inventory of Executive Function—Adult Version; DSM: DSM-5 Self-Rated Level 1 Cross-Cutting Symptom Measure—Adult; GPA: grade point average; NMBM: nonmedical use of benzodiazepine medication; NMPO: nonmedical use of prescription opioid medication; NMPS: nonmedical use of prescription stimulant medication; PROMIS: Patient-Reported Outcomes Measurement Information System; SRS: Sexual Risk Survey; * correlation is significant at the 0.05 level (2-tailed); ** correlation is significant at the 0.01 level (2-tailed).

**Table 4:** Sources for obtaining and most commonly used prescription opioids among lifetime nonmedical prescription opioid users.

Sources	College in Southeast (*n* = 25)	College in Northeast (*n* = 67)	All Colleges (*n* = 92)
Friends	10 (40.0%)	18 (26.9%)	28 (30.4%)
Dentist	3 (12.0%)	14 (20.9%)	17 (18.5%)
Primary Care MD	3 (12.0%)	11 (16.4%)	14 (15.2%)
Bought Illegally	5 (20.0%)	9 (13.4%)	14 (15.2%)
Parents	5 (20.0%)	8 (11.9%)	13 (14.1%)
Urgent Care	3 (12.0%)	6 (9.0%)	9 (9.8%)
Stole	4 (16.0%)	3 (4.5%)	7 (7.6%)
Emergency Room	1 (4.0%)	5 (7.5%)	6 (6.5%)
Orthopedic MD	1 (4.0%)	5 (7.5%)	6 (6.5%)
Other	1 (4.0%)	2 (3.0%)	3 (3.3%)
Specialist (Other)	1 (4.0%)	1 (1.5%)	2 (2.2%)
Pain Management Specialist	0 (0.0%)	1 (1.5%)	1 (1.1%)
**Most Common Prescription Opioids Used Nonmedically**			
Hydrocodone (e.g., Vicodin, Lorcet)	9 (36.0%)	16 (23.9%)	25 (27.2%)
Immediate Release Oxycodone (e.g., Roxicet, Roxicodone, Percocet)	8 (32.0%)	16 (23.9%)	24 (26.1%)
Codeine (e.g., Tylenol 3 and 4)	5 (20.0%)	17 (25.4%)	22 (23.9%)
Extended-Release Oxycodone (e.g., Oxycontin)	3 (12.0%)	10 (14.9%)	13 (14.1%)
Tramadol (Ultram, Tramal)	2 (8.0%)	5 (7.5%)	7 (7.6%)
Morphine (e.g., MS Contin, Oramorph)	0 (0.0%)	3 (4.5%)	3 (3.3%)
Hydromorphine (e.g., Dilaudid)	0 (0.0%)	3 (4.5%)	3 (3.3%)
Fentanyl (e.g., Duragesic, Actiq)	0 (0.0%)	3 (4.5%)	3 (3.3%)
Buprenorphine (e.g., Suboxone, Subutex)	0 (0.0%)	3 (4.5%)	3 (3.3%)
Methadone	0 (0.0%)	3 (4.5%)	2 (2.2%)
Oxymorphone (e.g., Opana, Numorphan)	1 (4.0%)	1 (1.5%)	2 (2.2%)

## Data Availability

The datasets analyzed for the current study are available from the corresponding author on reasonable request.

## Supplementary Materials

The following are available online at https://www.mdpi.com/article/10.3390/pharmacy9020106/s1: Table S1. Lifetime prevalence of nonmedical prescription opioid use by predictor variables; Table S2. Motives for nonmedical prescription opioid use of lifetime users.
