# Supplementary material for: Nonmedical Prescription Opioid Use among a Sample of College Students: Prevalence and Predictors
Source: Pharmacy (Basel). 2021 May 28;9(2):106. doi: 10.3390/pharmacy9020106 (PMC8167772; doi:10.3390/pharmacy9020106)
Supplement: Supplementary file 1 [file pharmacy-09-00106-s001.zip › Supplementary Table S1.pdf]

**Supplementary Table S1.** Lifetime prevalence of nonmedical prescription opioid use by predictor variables.

| Predictors | beta | SE | aOR | 95% LB | 95% UB | two-tailed p-value |
|---|---|---|---|---|---|---|
| Age | 0.131 | 0.216 | 1.140 | 0.746 | 1.743 | .545 |
| Male Sex | 0.190 | 0.187 | 1.210 | 0.839 | 1.745 | .308 |
| Student of Color | −0.412 | 0.448 | 0.663 | 0.275 | 1.595 | .359 |
| Hispanic or Latinx | 0.405 | 1.269 | 1.499 | 0.125 | 18.031 | .750 |
| GPA | −0.304 | 0.240 | 0.738 | 0.461 | 1.182 | .206 |
| Year in College | 0.043 | 0.201 | 1.044 | 0.704 | 1.546 | .831 |
| Member of Fraternity or Sorority | 0.332 | 0.300 | 1.394 | 0.774 | 2.508 | .268 |
| Alcohol | 0.575 | 0.102 | 1.777 | 1.455 | 2.171 | **<.001** |
| Marijuana Use | −0.078 | 0.443 | 0.925 | 0.388 | 2.206 | .861 |
| Nonmedical Use of Benzodiazapine Medication | 1.911 | 0.305 | 6.758 | 3.718 | 12.284 | **<.001** |
| Nonmedical Use of Prescription Stimulant Medication | 1.019 | 0.119 | 2.770 | 2.196 | 3.495 | **<.001** |
| Metacognition | −0.120 | 0.007 | 0.887 | 0.875 | 0.899 | **<.001** |
| Behavioral Regulation | 0.258 | 0.131 | 1.294 | 1.002 | 1.673 | **.049** |
| Depression | 0.368 | 0.037 | 1.444 | 1.343 | 1.553 | **<.001** |
| Anxiety | −0.186 | 0.057 | 0.830 | 0.743 | 0.927 | **.001** |
| Pain Behavior | 0.006 | 0.046 | 1.006 | 0.920 | 1.100 | .902 |

*Notes*. Analytic sample of predictor models was *n*=847; Missing data handled with FIML.

aOR = adjusted odds ratio; LB = lower bound; SE = standard error; UB = upper bound.
