# Supplementary material for: Nonmedical Prescription Opioid Use among a Sample of College Students: Prevalence and Predictors
Source: Pharmacy (Basel). 2021 May 28;9(2):106. doi: 10.3390/pharmacy9020106 (PMC8167772; doi:10.3390/pharmacy9020106)
Supplement: Supplementary file 1 [file pharmacy-09-00106-s001.zip › Supplementary Table S2.pdf]

Supplementary Table S2. Motives for Nonmedical Prescription Opioid Use of Lifetime Users

**Main Motives**	**Total *n***	**Never**	**Hardly Ever**	**Sometimes**	**Often**	**Very Often**
To relieve physical pain?	80	29 (36.3%)	18 (22.5%)	21 (26.3%)	7 (8.8%)	5 (6.3%)
Because it's fun?	79	37 (46.8%)	18 (22.8%)	14 (17.7%)	6 (7.6%)	4 (5.1%)
Because it gives you a pleasant feeling?	79	41 (51.9%)	11 (13.9%)	10 (12.7%)	8 (10.1%)	9 (11.4%)
To get high?	80	42 (52.5%)	9 (11.3%)	14 (17.5%)	10 (12.5%)	5 (6.3%)
Because you like the feeling?	80	44 (55.0%)	11 (13.8%)	10 (12.5%)	6 (7.5%)	9 (11.3%)
To relax or relieve tension?	79	44 (55.7%)	13 (16.5%)	11 (13.9%)	6 (7.6%)	5 (6.3%)
To experiment, see what it's like?	79	44 (55.7%)	14 (17.7%)	9 (11.4%)	8 (10.1%)	4 (5.1%)
Because it helps you sleep?	79	45 (57.0%)	13 (16.5%)	14 (17.7%)	3 (3.8%)	4 (5.1%)
To decrease anxiety?	79	45 (57.0%)	14 (17.7%)	9 (11.4%)	6 (7.6%)	5 (6.3%)
Because it helps you when you feel depressed or nervous?	78	47 (60.3%)	13 (16.7%)	8 (10.3%)	7 (9.0%)	3 (3.8%)
For pain related to an injury?	79	49 (62.0%)	10 (12.7%)	12 (15.2%)	8 (10.1%)	0 (0.0%)
To forget about your problems?	79	50 (63.3%)	11 (13.9%)	6 (7.6%)	7 (8.9%)	5 (6.3%)
To forget your worries?	79	50 (63.3%)	11 (13.9%)	7 (8.9%)	6 (7.6%)	5 (6.3%)
For headaches?	80	51 (63.7%)	9 (11.3%)	14 (17.5%)	4 (5.0%)	2 (2.5%)
To cheer up when you're in a bad mood?	78	51 (65.4%)	13 (16.7%)	5 (6.4%)	3 (3.8%)	6 (7.7%)
Because it's exciting?	79	54 (68.4%)	11 (13.9%)	3 (3.8%)	7 (8.9%)	4 (5.1%)
For muscle aches?	79	54 (68.4%)	13 (16.5%)	6 (7.6%)	5 (6.3%)	1 (1.3%)
Because it helps you enjoy a party?	80	55 (68.8%)	10 (12.5%)	10 (12.5%)	3 (3.8%)	2 (2.5%)
For pain conditions such as menstrual cramps, joint problems, or arthritis?	79	55 (69.6%)	7 (8.9%)	7 (8.9%)	6 (7.6%)	4 (5.1%)
Because it makes social gatherings more fun?	79	56 (70.9%)	8 (10.1%)	9 (11.4%)	4 (5.1%)	2 (2.5%)
To be sociable?	80	60 (75.0%)	9 (11.3%)	7 (8.8%)	2 (2.5%)	2 (2.5%)
To celebrate a special occasion with friends?	79	60 (75.9%)	7 (8.9%)	7 (8.9%)	2 (2.5%)	3 (3.8%)
Because you feel more self-confident or sure of yourself?	79	60 (75.9%)	7 (8.9%)	7 (8.9%)	3 (3.8%)	2 (2.5%)
Because it improves parties and celebrations?	79	60 (75.9%)	9 (11.4%)	7 (8.9%)	1 (1.3%)	2 (2.5%)
For back or neck problems?	78	60 (76.9%)	10 (12.8%)	6 (7.7%)	0 (0.0%)	2 (2.6%)
Because it is safer than street drugs?	79	66 (83.5%)	5 (6.3%)	5 (6.3%)	2 (2.5%)	1 (1.3%)
To counteract the effects of other drugs?	79	67 (84.8%)	6 (7.6%)	4 (5.1%)	1 (1.3%)	1 (1.3%)
Because you are addicted?	78	68 (87.2%)	6 (7.7%)	2 (2.6%)	0 (0.0%)	2 (2.6%)
